# Expression of Glut-1, HIF-1α, PI3K and p-Akt in a case of ceruminous adenoma

**DOI:** 10.1186/1758-3284-4-18

**Published:** 2012-05-02

**Authors:** Wan-Qin Shen, Ke-Jia Cheng, Yang-Yang Bao, Shui-Hong Zhou, Hong-Tian Yao

**Affiliations:** 1 Department of Otolaryngology, The First Affiliated Hospital, College of Medicine, Zhejiang University, Hangzhou, 310003, China; 2 Department of Pahtology, The First Affiliated Hospital, College of Medicine, Zhejiang University, Hangzhou, 310003, China

**Keywords:** Ceruminous adenoma, External auditory canal, Glucose transporter 1

## Abstract

**Objectives:**

Ceruminous adenoma of the external auditory canal (EAC) is a rare type of tumour that is diagnosed histologically. However, the clinical behaviour of these tumours remains controversial. Here, we report a case of ceruminous adenoma of the EAC and expression of a hypoxia marker.

**Case report:**

A 78-year-old man presented with a 6-month history of recurrent otorrhoea in the right ear. Surgery was performed by the transmeatal approach with total removal of the mass. Histopathology revealed a ceruminous adenoma. Tumour cells were positive for CK, S-100 protein, Glut-1, HIF-1α, PI3K and p-Akt. There was no evidence of recurrence at last follow-up 27 months after the operation.

**Conclusions:**

Ceruminous adenoma of the EAC is a rare tumour. The treatment of choice is wide local excision with clear margins. To our knowledge, this is the first report of Glut-1 expression and the PI3K/Akt pathway in ceruminous adenoma of the EAC.

## Background

Ceruminous adenoma of the external auditory canal (EAC) is a rare type of tumour. According to the classification of Mills et al. [1], this is a benign lesion and only approximately 150 cases of ceruminous adenoma at this level have been reported worldwide to date [2]; consequently, surgeons and pathologists have little experience with these neoplasms. Due to the limited number of cases, there has been considerable confusion regarding the diagnosis, nomenclature, and behaviour of ceruminous gland neoplasms [3]. Most cases follow a benign clinical course, and surgical resection with margins free of neoplasm is typically curative [1,2]. Some authors have suggested that histological examination of benign tumours of the EAC cannot predict biological behaviour and that all tumours should therefore be treated as potentially malignant [4]. Immunohistochemical staining for proteins such as CK5/6 and S-100 is an auxiliary method that has contributed to the differential diagnosis of ceruminous gland tumours [1,5].

The growth of tumours is highly dependent on glucose as the major energy source. Overexpression of glucose transporter 1 (Glut-1) has been demonstrated in many types of human tumour, including some benign tumours, such as benign salivary gland tumours [6,7], haemangiomas [8] and nerve sheath tumours [9]. This has also been interpreted as an adaptation to intermittent hypoxia, which occurs as a tumour outgrows its blood supply. In addition to its role as a glucose transporter, Glut-1 is known to play an important role in the cellular response to hypoxia, as a downstream target of hypoxia-inducible factor-1α (HIF-1α). The HIF complex then binds to hypoxia-responsive elements (HRE) in target genes and activates their transcription. In addition to proline hydroxylation, other regulatory pathways, including the phosphatidylinositol 3-kinase (PI3K)/Akt pathway, have been implicated in the control of HIF-1α protein expression and Glut-1 expression [10,11].

Here, we present a case of ceruminous adenoma in the EAC expressing Glut-1, HIF-1α, PI3K and p-Akt, which to our knowledge has not been reported previously in ceruminous gland tumours.

## Case report

**Figure 1:**
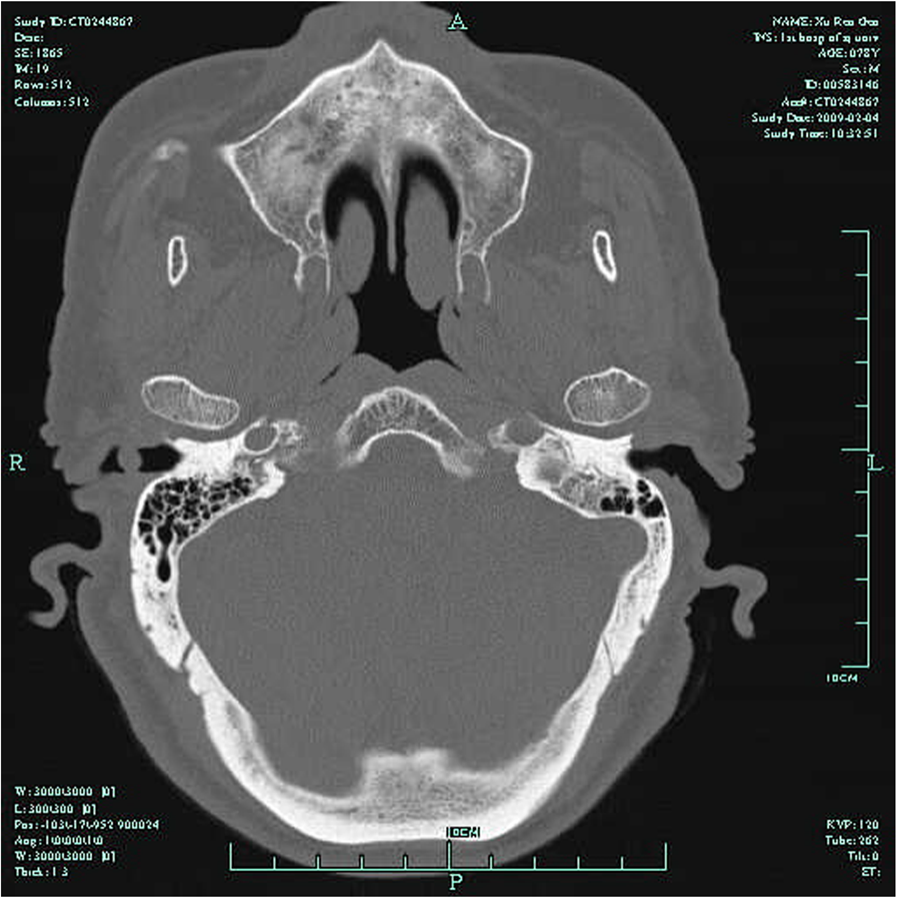
CT showed a 0.5×0.5cm soft lesion in the right EAC with no signs of bone destruction.

**Figure 2:**
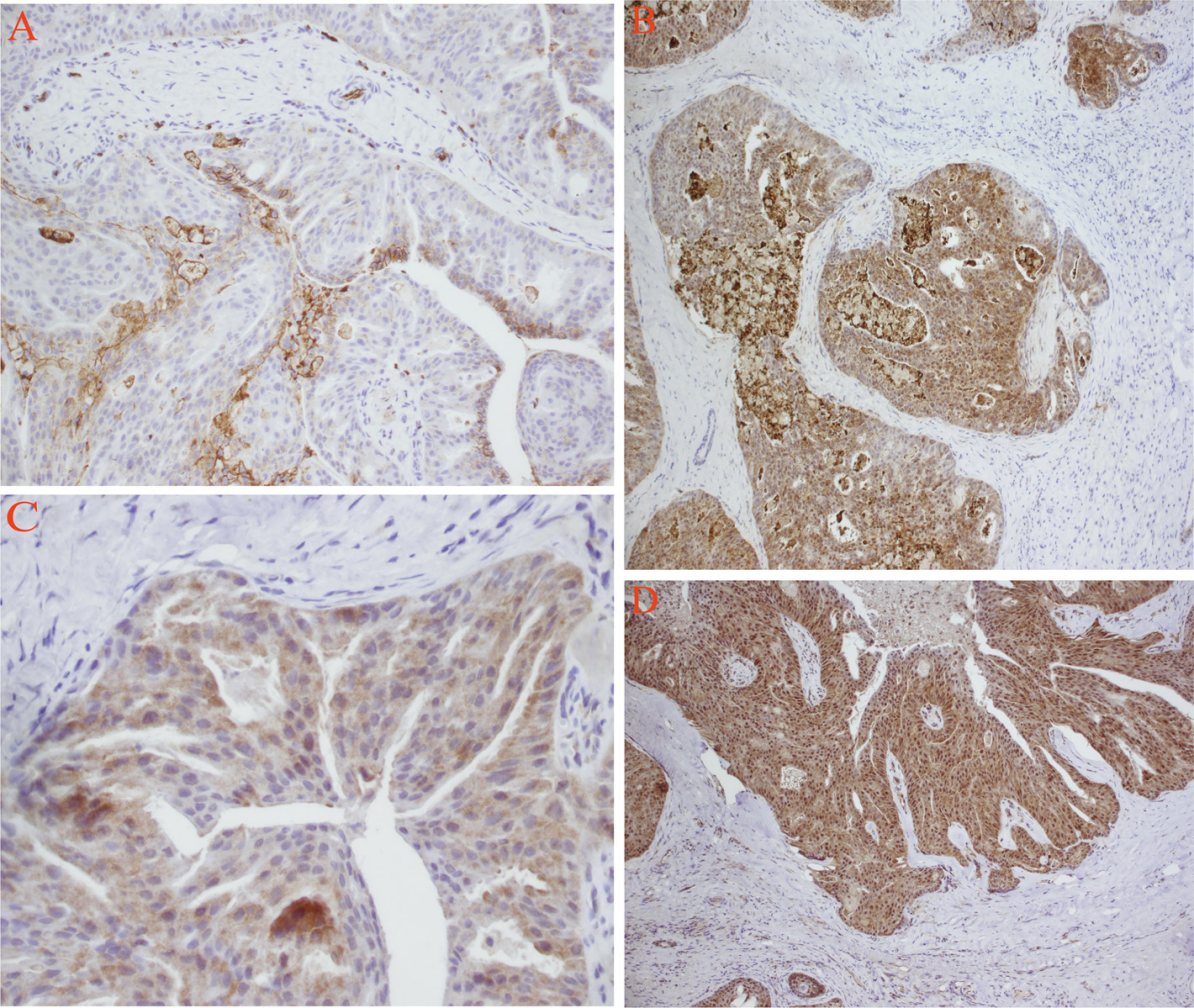
**Immunohistochemistry analyses for the expression of Glut-1, HIF-1α, PI3K and p-Akt.** The tumor cells were positive for Glut-1(**A**) (EliVision×20), HIF-1α(**B**) (EliVision×10), PI3K(**C**) (EliVision×40) and p-Akt(**D**) (EliVision×10).

A 78-year-old man presented with a 6-month history of recurrent otorrhoea in the right ear. He reported no pain, hearing loss, tinnitus or vertigo. He had no history of trauma, surgery or of wearing a hearing aid. Physical examination showed a pink, smooth mass measuring 0.5  ×  0.7 cm on the outer part of the right EAC. Palpation of the mass revealed no tenderness. A computed tomography (CT) examination showed a soft lesion measuring 0.5 × 0.5 cm in the right EAC with no sign of bone destruction, but the radiologist made a diagnosis of infection and did not suspect a tumour (Figure 1). Surgery was performed by the transmeatal approach with total removal of the mass. Histopathology revealed that the tumour cells were arranged in a glandular nest, similar to normal ceruminous glands. The cells were growing actively, and a diagnosis of adenoma of the ceruminous gland was made. Immunohistochemical analyses for the expression of vimentin, cytokeratin (CK), alpha-smooth muscle actin (α-SMA), desmin, S-100 protein, Glut-1, HIF-1α, PI3K and p-Akt were performed in the tissue sample using an EliVision plus IHC Kit (Maixin Biological, Fuzhou, China). The tumour cells were positive for CK, S-100 protein, Glut-1, HIF-1α, PI3K and p-Akt (Figure 2), but negative for all other markers examined.

At final follow-up, 27 months after the operation, there was no evidence of recurrence.

## Discussion and Conclusions

Ceruminous gland tumours are uncommon lesions arising from the EAC, the behaviour of which is still unclear. In contrast to the view that most ceruminous gland tumours can be classified accurately histologically as either benign or malignant [12], some authors have suggested that all ceruminous gland tumours should be regarded as potentially malignant [4,12]. Complete surgical resection is the treatment of choice and is associated with long-term tumour-free survival. Recurrence on follow-up may occur [5]. However, there has been no recurrence in our case after 27 months of follow-up.

The differential diagnosis of ceruminous adenoma includes ceruminous adenocarcinoma, neuroendocrine adenoma of the middle ear, parotid pleomorphic adenoma, meningioma and paraganglioma [5]. The results of immunohistochemical analyses are identical to those of normal cerumen glands, supporting the histogenesis of this benign neoplasm [1,5]. All adenomas have a dual cell population, composed of inner luminal epithelial cells subtended by basal myoepithelial cells adjacent to the basement membrane. These cell populations are accentuated in immunohistochemical studies; the luminal cells are strongly immunoreactive for cytokeratin 7, whereas the basal myoepithelial cells are strongly and diffusely reactive for S-100 protein [1,5].

In the present case, the neoplastic cells were richer, showed active growth, and were positive for CK and S-100 protein, suggesting possible roles of these proteins in the development of these cells. Glut-1 overexpression in tumour tissue is involved in the cellular response to hypoxia, as a downstream target of HIF-1α [8,13]. Glut-1 upregulation and subsequent overexpression of Glut-1 receptors on the plasma membrane of various tumour cells are thought to allow escape from the apoptosis-inducing effects of hypoxic environments [8]. HIF-1α modulates the expression of many genes involved in various processes, such as angiogenesis and pH regulation, and Glut-1 expression has a central role in this process. PI3K/AKT signalling plays an important role in the adaptive response of tumour cells to hypoxia [14,15]. To some extent, under the control of AKT, information on AKT activation status could substantially add to the predictive potential of endogenous tumour markers (Glut-1, HIF-1α) [10,11,14]. Glut-1, HIF-1α, PI3K and p-Akt expression in neoplastic cells may be important in the development of a ceruminous adenoma.

In conclusion, ceruminous adenoma of the EAC is rare type of tumour that is diagnosed histologically. The treatment of choice is wide local excision with clear margins. Glut-1, HIF-1α, PI3K and p-Akt expression in neoplastic cells may be important for the development of ceruminous adenoma.

## Consent

Written informed consent was obtained from the patient for publication of this Case report and any accompanying images. A copy of the written consent is available for review by the Editor-in-Chief of this journal.

## Abbreviations

EAC: External auditory canal; Glut-1: Glucose transporter-1; HIF-1α: Hypoxia-inducible factor 1-α; HRE: Hypoxia-responsive elements; PI3K/Akt: Phosphatidylinositol 3-kinase /Akt pathway; CK: Cytokeratin; α-SMA: Alpha-smooth muscle actin.

## Competing interests

The authors declare that they have no competing interests.

## Authors’ contributions

Wan-Qin Shen designed the manuscript and participated in nursing. Ke-Jia Cheng participated in the surgery and data collection. Yang-Yang Bao participated in data collection. Shui-Hong Zhou performed the surgery and drafted the manuscript, revised the article for important intellectual content. Hong-Tian Yao carried out the histological examination and took pathological figures. All authors read and approved the final manuscript.
